# Automated 3D EBSD for metallic powders

**DOI:** 10.1016/j.mex.2018.06.001

**Published:** 2018-06-11

**Authors:** Caitlin Walde, Roger Ristau, Danielle Cote

**Affiliations:** a Worcester Polytechnic Institute, United States; b University of Connecticut, United States

**Keywords:** Automated 3D-EBSD for metallic powders using a Xe P-FIB, Additive manufacturing, Powder metallurgy, Metallography, Characterization, Electron microscopy, Serial sectioning, Three-dimensional microscopy, 3D microscopy, 3D sectioning

## Abstract

Metallic powders are commonly used in additive manufacturing processes. While their post-process consolidated properties are widely studied, there is little research on the properties of the powders prior to consolidation. Understanding the powder characteristics before use in additive manufacturing processes could lead to fine-tuning properties of additively manufactured materials. The three-dimensional grain structure of metals can be useful in predicting their properties and microstructure. Powder particles are much smaller and more difficult to fixture and polish than their bulk counterparts, hence typical protocols are difficult to use when serially sectioning them. This method describes a recommendation as to how to fixture, mill, and image metallic powder particles using a Xe P-FIB to mill and take EBSD measurements. It is based on milling and imaging techniques used for bulk materials, but with the specific advantage of how to fixture the powder sample. Our modifications include:

• the method of fixturing the specimen to the holder.
• the method of protecting the sample during milling.

## Graphical abstract

**Figure:**
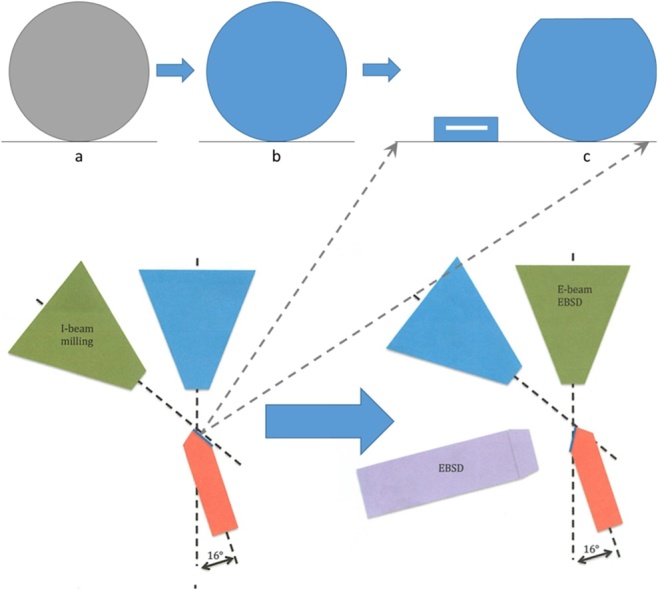

Specifications Table

**Table:** 

Subject area	Materials Science
More specific subject area	Powder metallurgy
Method name	Automated 3D-EBSD for metallic powders using a Xe P-FIB
Name and reference of original method	Uchic, Michael D., et al. “3D microstructural characterization of nickel superalloys via serial-sectioning using a dual beam FIB-SEM.” *Scripta Materialia* 55.1 (2006): 23–28.
Resource availability	FEI Helios Xenon Plasma FIB (P-FIB) Avizo (or other 3D reconstruction software) 54° Pre-tilted EBSD holder

## Method details

This method was developed to be used with an FEI Helios P-FIB, though it can easily be used in other instruments by adjusting the offset angles between the electron and ion beams as well as their beam current capabilities.

**Fig. 1:**
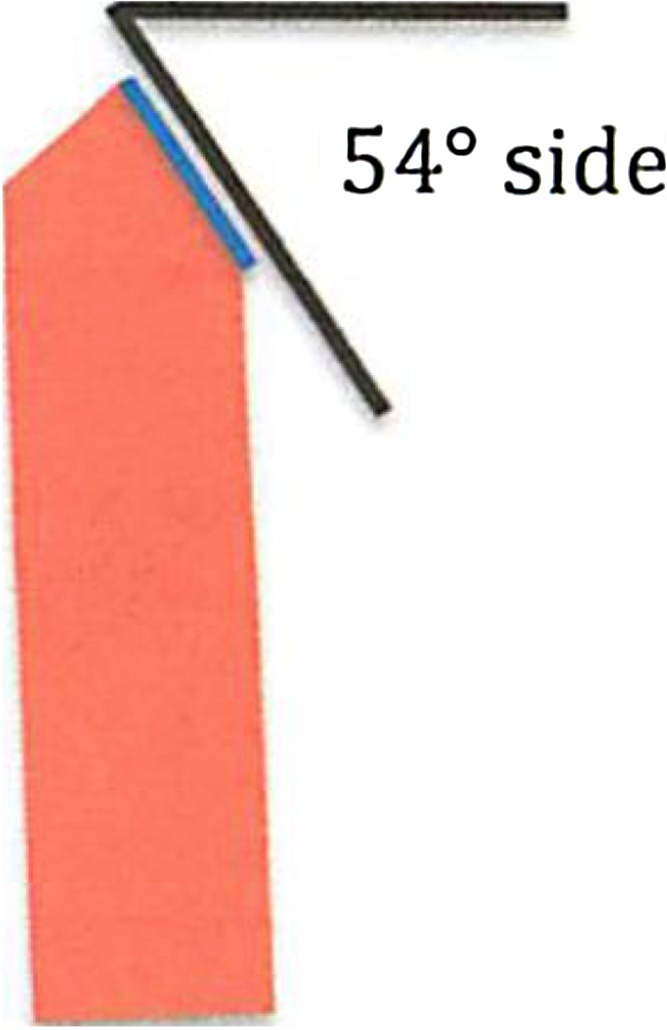
Diagram of pre-tilted holder, denoting the 54° side.

Similar to the original methods considered [[1], [2], [3], [4]], a 54° pre-tilted holder was used for this study (Fig. 1). Typically, a bulk material would be ground and polished before use in this type of study, however, powders bring unique challenges with regards to fixturing for polishing and evaluation. It is of interest to section a whole particle; however, if the powders are mounted and polished via traditional metallographic methods, this is not possible [5,6]. First attempts were made to distribute the powder on a piece of double-side Cu tape and a carbon dot, however the adhesive charged during imaging, causing the powder to move around. It was then attempted to embed to powder in a thin layer of carbon paint on an SEM stub. This method made it difficult to distinguish powder particles from other, random bumps in the layer. Additionally, this method off-gassed a considerable amount during milling, causing instabilities in the beam. After these attempts, the recommended method was successfully implemented.

**Fig. 2:**
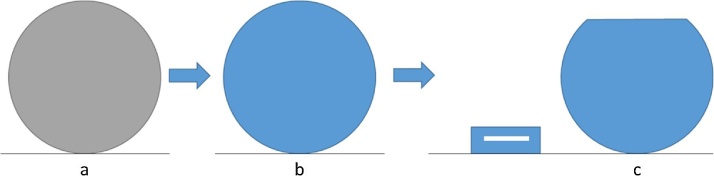
Ion beam view from milling position of a) particle, b) particle with Pt coating, c) particle with top removed and fiducial column.

This method entails cleaning the pre-tilted holder of all dust and other debris and loosely distributing powder on the 54° side of the pre-tilted holder (focusing near the top edge). The static forces are enough to holder the powder in place temporarily. Once the holder is in the FIB, the eucentric height is found as described in the original methods considered [[1], [2], [3], [4]], or as normally performed in the instrument. Once a particle i selected – based on user requirements for shape and size – and brought to the eucentric height, the Pt deposition in the FIB was used to secure the particle to the substrate. This was performed by using a Pt-Dep patterning circle with diameter 5 μm larger than that of the selected particle, with a z-depth of 1 μm (Fig. 2a and b). This was then performed 2 more times; once with the sample rotated +45° and again at −45°, to more effectively coat the sides of the particle. Together, these depositions secure the particle to the substrate, as well as provide a protective layer to retain surface finish during milling.

Typically, fiducials for automated imaging processes are made on the sample, near the site of milling and imaging. However, since this method is optimized for imaging of the entire powder particle, placing the fiducials on the particle is not possible. Therefore, a column of Pt with dimensions 10 × 10 × 5 μm was built near the desired powder particle (about 10 μm diagonally away from it), and fiducial markers were milled into the side and top (one for milling and EBSD, another for imaging and EBSD); see Fig. 2c.

As described in the originally considered methods [[1], [2], [3], [4]], the imaging parameters should be set up prior to beginning the automated sectioning process. In these methods, the sample is assumed already fairly polished, however with a whole powder particle, this is not the case. Therefore, a polished surface must be exposed on the particle. This was accomplished by simply milling off 1–2 μm of the particle (Fig. 2c) with the ion beam, followed by a subsequent, lower current, polishing step. This second step is typical of other FIB methods.

**Fig. 3:**
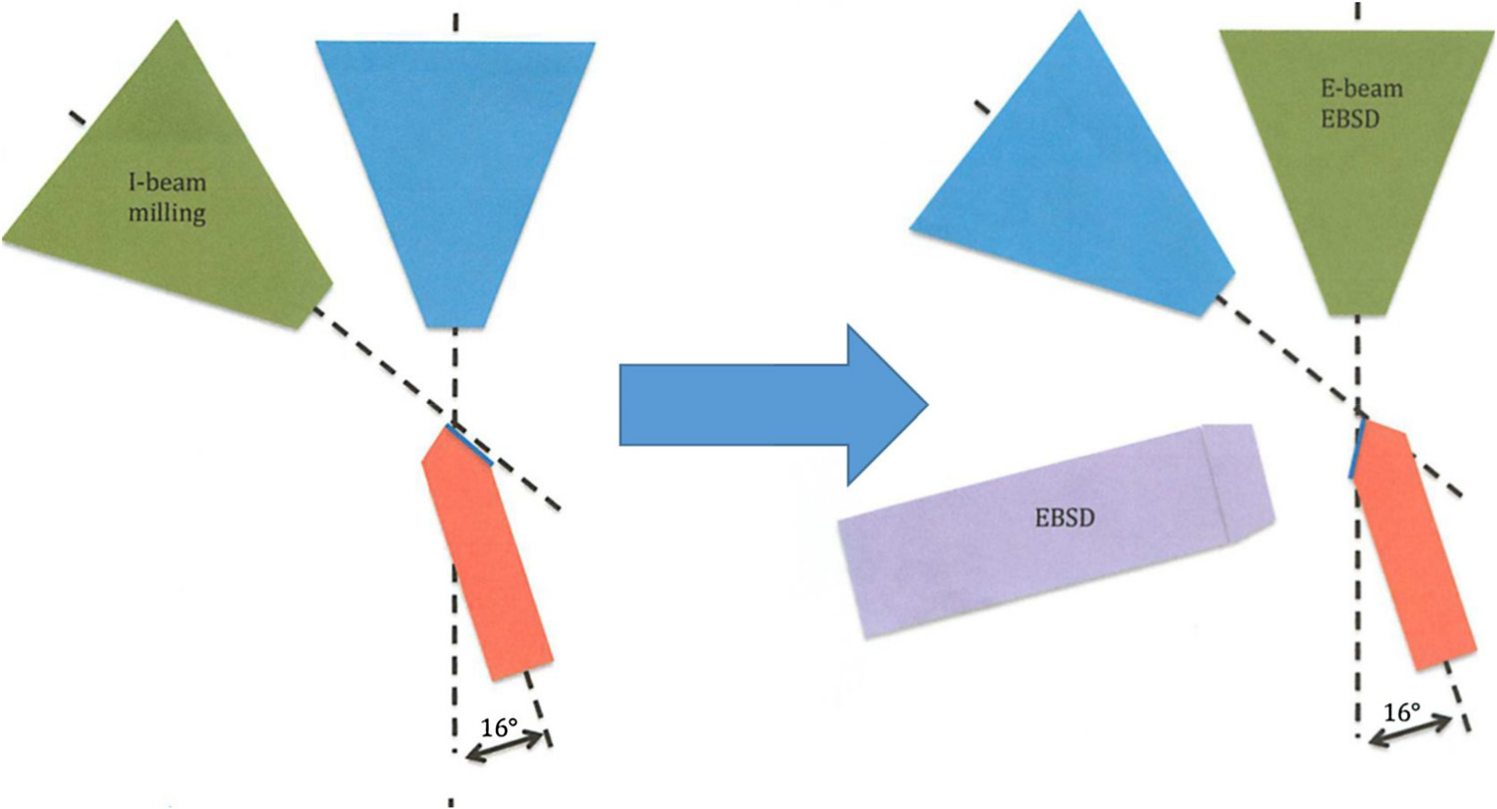
Overview of Milling (left) and EBSD (right) locations.

Automated imaging was then performed, similar to that described in the original methods [[1], [2], [3], [4]] and shown in Fig. 3, where the sample alternates between being milled by the ion beam and imaged by the electron beam.

Applications and results from this process can be seen elsewhere [5,6].
